# Antigen-affinity controls pre-germinal centser B cell selection by promoting Mcl-1 induction through BAFF receptor signaling

**DOI:** 10.1038/srep35673

**Published:** 2016-10-20

**Authors:** Felix M. Wensveen, Erik Slinger, Martijn HA van Attekum, Robert Brink, Eric Eldering

**Affiliations:** 1 Department of Experimental Immunology, Academic Medical Center, 1105AZ, Amsterdam, The Netherlands; 2 Immunology Division, Garvan Institute of Medical Research, NSW 2010, Darlinghurst, Australia

## Abstract

Upon antigen encounter, the responsive B cell pool undergoes stringent selection which eliminates cells with low B cell receptor (BCR) affinity. Already before formation of the germinal center, activated B cells of low-affinity are negatively selected in a process that is molecularly not well understood. In this study, we investigated the mechanism behind pre-GC affinity-mediated B cell selection. We applied affinity mutants of HEL antigen and found that rapidly after activation B cells become highly dependent on the cytokine BAFF. Moreover, expression of BAFF receptor CD268 is regulated in a BCR-affinity dependent fashion. High affinity responses via BAFF correlated with PI3K activation, which controlled expression of the pro-survival protein Mcl-1, and thereby increased survival. In the presence of excess BAFF, or in absence of the Mcl-1 antagonist Noxa, more low-affinity B cells survived the first two days after antigen encounter. This resulted in increased numbers of antigen-specific B cells of low affinity upon immunization and reduced the overall affinity of cells that contributed to the germinal center reaction. Our findings elucidate a crucial molecular pathway of B cell selection in the earliest phases of activation by identifying a novel link between BCR affinity and BAFF-R signaling towards Mcl-1.

The humoral immune response provides lasting protection against (re)-infection. Upon pathogen encounter, antigen-specific B cell clones are selected from a vast pool of cells, each one unique based on its antigen receptor. The minimal ligand-affinity of this B cell receptor (BCR) required for cell activation is relatively low and each pathogen therefore stimulates many cells^1^,^2^. To prevent sub-optimal B cells from consuming precious nutrients and cytokines, the antigen-responsive cell pool is subject to selection for only those cells with the highest specificity^3^. This process is most rigorous in the germinal center (GC), a structure which arises several days after antigen encounter^4^. Here, the overall antigen affinity of the responsive B cell pool is rapidly increased through active editing of the BCR via somatic hypermutation^3^,^5^. Cells of reduced affinity are eliminated via apoptosis in a Darwinian selection process that ensures only survival of the fittest clones^3^,^5^.

To ensure an efficient GC-reaction, the number of clones that is allowed to enter this structure must be restricted^6^. The activated B cell pool is therefore subject to antigen-affinity based selection from the earliest stages of B cell activation^7^. This selection appears to be independent of an intrinsic survival rheostat, but is driven by the competitive pressure of other activated B cell clones^1^. In absence of competing clones with a higher affinity, even cells of very low affinity are able to generate B cell responses of equal magnitude as high-affinity cells. However, when high- and low-affinity B cells are competing, high-affinity cells predominate in the antibody-producing cell pool upon immunization^1^. This system ensures that at all times a B cell response of the highest affinity is generated, independent of the initial affinity of the B cell pool.

CD4 T cell help^7^ plays an important role in affinity-based selection in the pre-GC stage. Within 6 hours after antigen recognition, activated B cells move to the border of B and T cell follicles^8^. B cells of reduced affinity take up less antigen than high-affinity cells, resulting in a reduced number of non-self peptides presented in MHC-II molecules to CD4 T cells^7^. Thus, high- and low-affinity B cells actively compete with each other for T cell-derived help. However, the nature of this help and whether T cell help is the only mechanism of pre-GC B cell selection, is currently unknown.

Previously, the Bcl-2 family of pro-and anti-apoptotic proteins was shown to be the key mediator of activated B cell survival^9^,^10^. Upon activation, B cells upregulate the pro-survival molecules Mcl-1 and Bcl-XL, whereas Bcl-2 expression is reduced^9^. Loss of even a single copy of the Mcl-1 gene in activated B cells results in a strong reduction of cell numbers^10^. Bcl-XL plays an essential survival role late in the B cell response, when plasmablasts leave the lymph node and home to the bone marrow^9^. Pro-survival members of the Bcl-2 family are antagonized by BH3 only proteins, such as Bim, Puma and Noxa^11^. Bim and Puma bind and inactivate all pro-survival Bcl-2 proteins and are therefore strong mediators of apoptosis. Deficiency of Bim or Puma prevents elimination of low-affinity cells in the GC, impairing affinity maturation^11^,^12^. Noxa is a weaker pro-apoptotic protein, because it only antagonizes A1 and Mcl-1. Loss of Noxa does not affect affinity maturation, but rather restricts the number of GC-seeding clones^6^. How Noxa mediates pre-GC selection on a molecular level is currently unknown.

Here we show that antigen-affinity positively correlates with the capacity to upregulate receptors for the pro-survival cytokine BAFF in the first days after B cell activation. High-affinity B cells show increased PI3K signaling in response to BAFF, resulting in enhanced stabilization of Mcl-1 protein and improved survival compared to low-affinity cells. In the presence of excess BAFF, or when B cells are deficient for Noxa, low-affinity cells comprise a much larger fraction of the antigen-responsive pool. Our findings shed new light on the earliest phases of B cell responses and may contribute to development of vaccines with a broader scope.

## Results

### BCR affinity differentially regulates expression of CD25 and CD268

To investigate the role of antigen affinity on the survival of B cells in the first days after activation, we used murine B cells expressing a transgenic antigen receptor (MD4) that recognizes Hen Egg Lysozyme (HEL). B cells were cultured in the presence of purified HEL, or HEL mutants carrying two (HEL2x) or three (HEL3x) substitutions, resulting in a 250 and 13.000 fold lower affinity for MD4 respectively^13^. When cultured in the presence of antigen alone, rapid activation-induced cell death occurred, which emphasized the requirement for a cofactor. Whereas LPS was the most potent stimulus for activation of B cells, from various cytokines implied in B cell survival, BAFF (Also known as BlyS) was shown to be the most efficient cofactor in this culture system (Supplementary Figure 1a). To study the effects of affinity on early B cell behavior we cultured cells therefore in the presence of low amounts of BAFF, and a concentration range of various HEL antigens.

**Figure 1:**
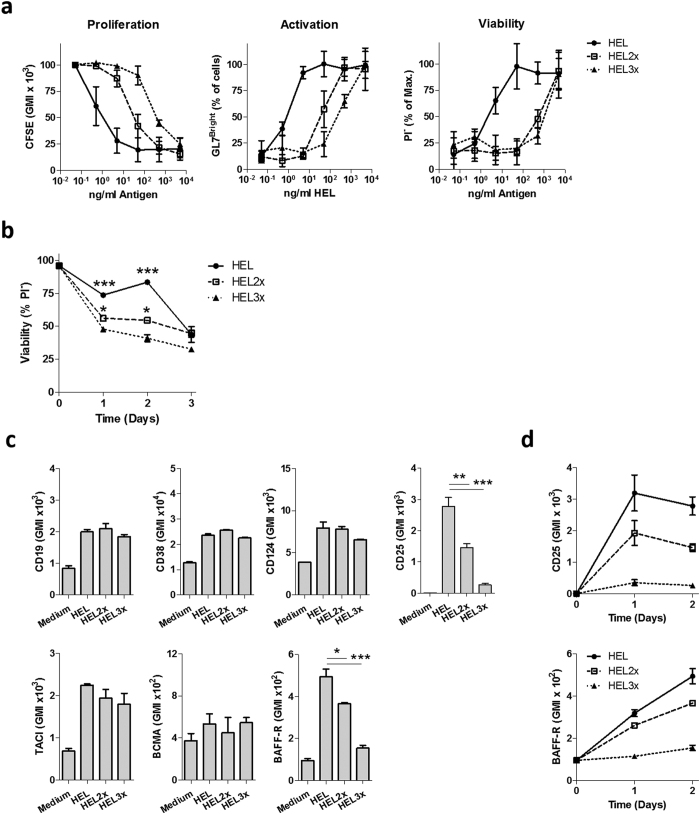
BCR affinity correlates with CD25 and BAFF-R induction. (**a**) HEL^TG^ B cells were stimulated for 48 h with 100 ng/ml HEL, HEL2x or HEL3x in combination with 100 ng/ml BAFF. Proliferation, activation and viability were assessed by CFSE dilution, GL7 induction and PI negativity respectively using flow cytometry (n = 3). (**b**) HEL^TG^ B cells were stimulated with 100 ng/ml HEL, HEL2x or HEL3x in combination with 100 ng/ml BAFF. Viability was followed over time by determining the PI negative fraction (n = 3). (**c**) HEL^TG^ B cells were stimulated with 100 ng/ml HEL, HEL2x or HEL3x in combination with 100 ng/ml BAFF. After 48 h, cell surface expression of indicated molecules was analyzed by flow cytometry (n = 3). (**d**) HEL^TG^ B cells were stimulated with 100 ng/ml HEL, HEL2x or HEL3x in combination with 100 ng/ml BAFF. Cell surface expression of CD25 and BAFF-R was followed over time by flow cytometry (n = 3). *p < 0.05, **p < 0.01, ***p < 0.001 (ANOVA with Bonferroni’s post-testing). GMI = Geometric Mean Intensity.

Proliferation and activation of B cells, as measured by CFSE dilution and GL7 upregulation respectively, correlated strictly with antigen concentrations. When using antigen of reduced affinity, higher concentrations were required to achieve similar effects (Fig. 1a, Supplementary Figure 1b). The EC50 for CFSE dilution (0.58, 24 and 256 ng/ml for HEL, HEL2x and HEL3x respectively) was comparable to that of GL7 induction (0.69, 34 and 213 ng/ml for HEL, HEL2x and HEL3x). In contrast, whereas the EC50 of survival was not significantly different from that of proliferation and activation for high-affinity stimulation (1.60 ng/ml), it was five to ten fold higher for HEL3x and HEL2x respectively (487 and 1003 ng/ml for HEL2x and HEL3x) (Fig. 1a, Supplementary Figure 1b). This indicates that low-affinity antigens are incapable of inducing a requisite factor for activated B cell survival, also under conditions that can fully activate B cells, which is not directly dependent on the level of proliferation.

Previously, we have shown in T cells that antigen-affinity closely correlates with IL-2Rα (CD25) expression, and potentiates sensitivity to the survival cytokine IL-2^14^,^15^. We hypothesized that B cell receptor affinity might similarly correlate with expression of a receptor for a pro-survival cytokine. Differences in survival following high- and low-affinity stimulation were only observed in the first two days after activation (Fig. 1b), marking the time frame in which this survival factor operates. At this time point, various receptors were induced in response to antigen-stimulation, but only BAFF-R (CD268) and IL-2Rα (CD25) correlated with antigen-affinity (Fig. 1c). Further analysis revealed that expression of the IL-2Rα subunit peaked at 24 hours after stimulation, whereas CD268 expression had not yet reached a maximum after 48 hours (Fig. 1d). BCMA and TACI, the two additional receptors that are able to bind BAFF were not regulated in an antigen-affinity dependent fashion (Fig. 1c). Thus, antigen affinity positively correlates with B cell survival in the first two days after activation and with induction of BAFF-R and IL-2Rα.

### BAFF, but not IL-2 mediates activated B cell survival in an antigen-dependent fashion

**Figure 2:**
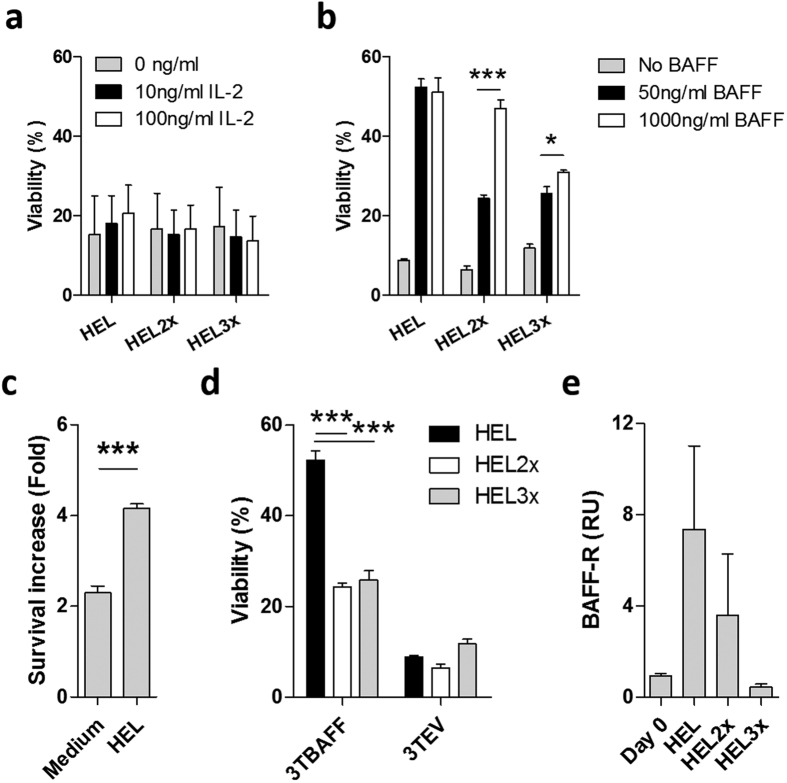
BAFF but not IL-2 controls early activated B cell survival. (**a,b**) HEL^TG^ B cells were stimulated with 100 ng/ml HEL, HEL2x or HEL3x in combination with (**a**) 10 ng/ml or 100 ng/ml of IL-2 or (**b**) with 50 ng/ml or 1000 ng/ml of BAFF and viability (PI^−^) was analyzed after 48 h by flow cytometry (n = 3–4). (**c**) HEL^TG^ B cells were left untreated (Medium) or stimulated with 100 ng/ml HEL in the presence or absence of 100 ng/ml BAFF and viability (PI^−^) was assessed after 48 h by flow cytometry. Shown is the fold increase in the percentage of viable cells after BAFF stimulation compared to cells cultured in absence of BAFF (n = 3). (**d**) HEL^TG^ B cells were stimulated with 100 ng/ml HEL, HEL2x or HEL3x and cultured on irradiated 3T3 cells transfected with a membrane-bound form of human BAFF (3TBAFF) or empty vector (3TEV). Viability (PI^−^) was assessed after 48 h by flow cytometry (n = 3). (**e**) HEL^TG^ B cells were stimulated with 100 ng/ml HEL, HEL2x or HEL3x in combination with 100 ng/ml BAFF. BAFF-R mRNA levels were analyzed after 48 h by qPCR (n = 3). Values show means ± sem. Shown is relative induction of gene expression compared to Day 0. *P < 0.05, **P < 0.01, ***P < 0.001 (Student’s *t*-test and ANOVA with Bonferroni’s post-testing).

To investigate the impact of BAFF and IL-2 on survival, activated B cells were stimulated in the presence of either cytokine and viability was assessed after two days. Even high concentrations of IL-2 failed to improve survival (Fig. 2a). In contrast, low concentrations of BAFF achieved a strong survival increase of activated B cells, which was most prominent after high-affinity stimulation (Fig. 2b). When high concentrations of BAFF were used, low- but not high-affinity B cells showed a further increase of viability, indicating that BAFF becomes limiting for survival of low-affinity cells. As reported previously^16^, BAFF promoted survival of both resting and antigen-stimulated cells. However, the increase in survival mediated by BAFF was significantly higher for activated cells, indicating that antigen-encounter makes B cells more dependent on this cytokine to sustain viability (Fig. 2c).

To investigate the impact of BAFF on activated B cell survival in a model independent of recombinant cytokine, 3T3 cells were generated that express a form of human BAFF on their membrane that cannot be cleaved off (Supplementary Figure 1c–e). HEL^TG^ B cells were cultured on these cells in the presence of high- or low-affinity antigen. 3T3 cells expressing BAFF (3TBAFF) increased B cell survival significantly over cells cultured on 3T3 cells transduced with an empty vector (3TEV). Notably, high-affinity B cells showed significantly better survival than low-affinity cells when cultured in the presence of 3TBAFF cells (Fig. 2d).

To investigate whether the BAFF receptor is induced through transcriptional upregulation, we performed qPCR analysis of BAFF-R mRNA in HEL^TG^ B cells stimulated *in vitro* with HEL mutants. This showed that BAFF-R transcription was induced in an affinity-dependent fashion (Fig. 2e). In summary, activated B cells induce expression of the BAFF-R in an affinity-dependent fashion, which sensitizes cells to BAFF-mediated survival.

### BCR affinity primes cells for BAFF-induced Mcl-1 protein upregulation

**Figure 3:**
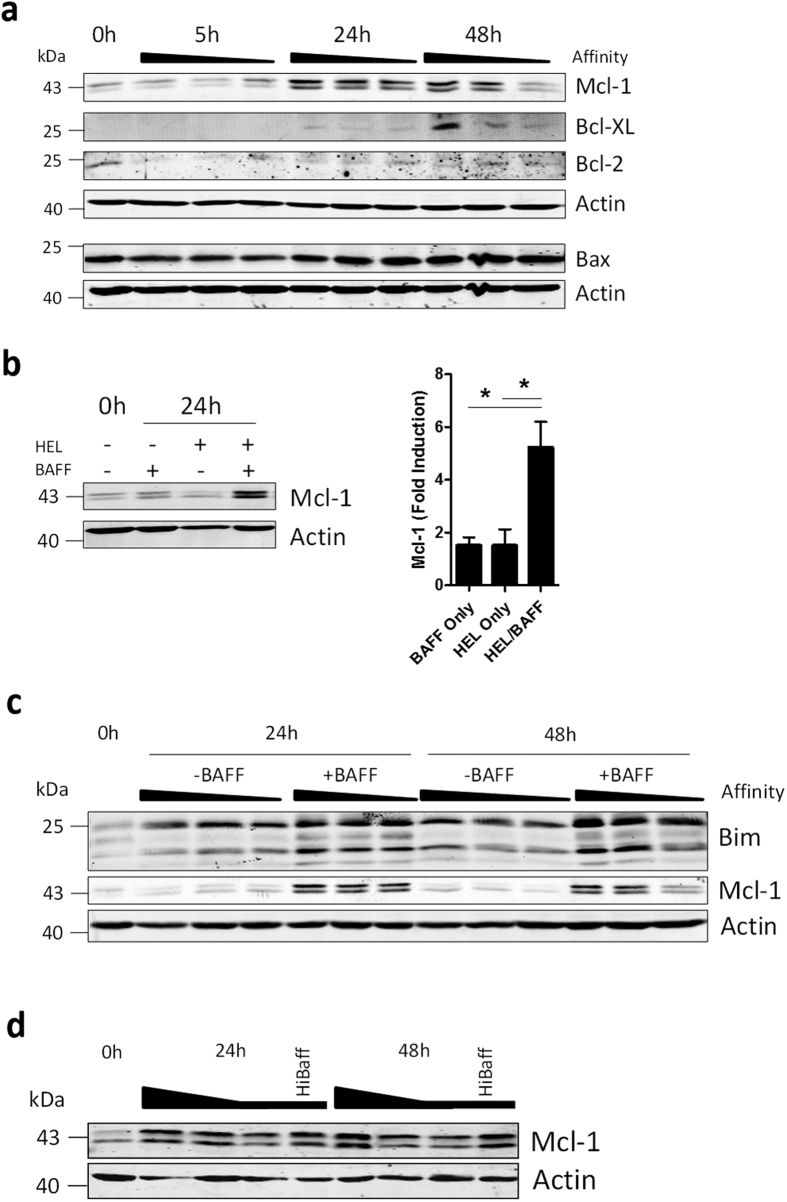
BAFF controls Mcl-1 protein levels in a BCR affinity-dependent manner. (**a**) HEL^TG^ B cells were stimulated with 100 ng/ml HEL, HEL2x or HEL3x in the presence 100 ng/ml BAFF. (n = 8) (**b**) HEL^TG^ B cells were stimulated for 24 h with 100 ng/ml HEL, 100 ng/ml BAFF or both (n = 3) (**c**) HEL^TG^ B cells were stimulated with 100 ng/ml HEL, HEL2x or HEL3x in the presence or absence of 100 ng/ml BAFF (n = 3). Protein levels were quantified using densitometry and mean levels normalized for β-actin are shown in comparison to T = 0. (**d**) HEL^TG^ B cells were stimulated with 100 ng/ml HEL, HEL2x or HEL3x in the presence 100 ng/ml BAFF or with 100 ng/ml HEL3x in combination with 500 ng/ml BAFF (HiBaff). Total cell lysates were then probed by western blot for the indicated proteins. β-Actin was used as a loading control. Affinity ‘scale bars’ correspond with high (HEL), middle (HEL2x) or low (HEL3x) affinity ligands used for stimulation (n = 3). Values show means ± sem. *P < 0.05 (Student’s *t*-test and ANOVA with Bonferroni’s post-testing).

Survival of activated B cells depends on the differential regulation of several members of the Bcl-2 family of pro-and anti-apoptotic proteins. Antigen recognition is associated with upregulation of the pro-survival proteins Bcl-XL and Mcl-1, whereas Bcl-2 is down-modulated^10^. Especially loss of Mcl-1 is associated with rapid cell death of activated B cells^10^. Therefore, we analyzed protein expression of these three molecules in the context of antigen-affinity dependent activation. Stimulation with HEL proteins resulted in a rapid downregulation of Bcl-2. In contrast, we observed a strong induction of Mcl-1 which correlated positively with BCR affinity, especially after 48 hours (Fig. 3a, Supplementary Figure 2a). Bcl-XL levels were also induced, but at a later time point after antigen stimulation. These findings prompted us to further investigate antigen affinity-mediated Mcl-1 induction.

BAFF has been shown to induce Mcl-1 in resting B cells and promote their survival^17^. Indeed, we observed that BAFF or antigen alone resulted in a modest increase of Mcl-1 levels. Reminiscent of the induction of BAFF-R in activated B cells, we observed a much stronger induction of Mcl-1 in the presence of both antigen and BAFF (Fig. 3b). Importantly, Mcl-1 levels correlated strongly with antigen-affinity in the presence of BAFF and were much lower when BAFF was omitted (Fig. 3c, Supplementary Figure 2b). Protein levels of pro-apoptotic Bcl-2 family member Bim, which have previously been shown to increase in response to cytokine deprivation^18^, were not reduced in the presence of BAFF (Fig. 3c). Moreover, Mcl-1 levels could be increased in cells stimulated with low-affinity antigen through addition of extra BAFF (Fig. 3d, Supplementary Figure 2c). In summary, the strength of BCR triggering is transduced via BAFF signaling to adjust Mcl-1 protein levels.

### BCR affinity promotes B cell survival by PI3K- Akt-GSK3β signaling in response to BAFF

**Figure 4:**
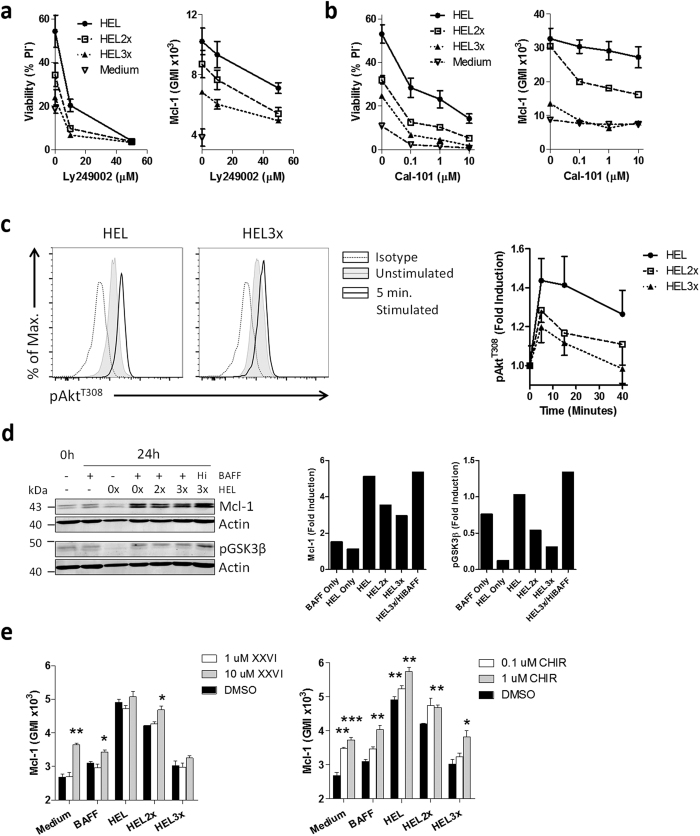
BCR affinity promotes BAFF induced PI3K signaling. (**a,b**) HEL^TG^ B cells were left unactivated (Medium), or stimulated with 100 ng/ml BAFF alone (BAFF only) or in combination with 100 ng/ml HEL, HEL2x or HEL3x in the presence or absence of (**a**) the Pan-PI3K inhibitor Ly249002 or (**b**) the PI3Kδ inhibitor CAL-101. After 48 h viability and Mcl-1 protein levels were assessed by flow cytometry (n = 3). (**c**) HEL^TG^ B cells were stimulated with 100 ng/ml BAFF in combination with 100 ng/ml HEL, HEL2x or HEL3x. After 48 h, cells were deprived from stimuli for 3 h, followed by stimulation with 500 ng/ml BAFF. pAkt^T308^ levels were measured by intracellular flow cytometry at the indicated time points (n = 3). (**d**) HEL^TG^ B cells were activated with the indicated stimuli. Mcl-1 and pGSK3β levels were determined by western blot. β-actin was used as a loading control. Quantification shows fold induction over the signal on day 0, normalized for β-actin. (**e**) HEL^TG^ B cells were left inactivated (Medium), or stimulated with 100 ng/ml BAFF alone (BAFF only) or in combination with 100 ng/ml HEL, HEL2x or HEL3x in the presence of solvent only (DMSO)or the GSK3 inhibitors XXVI or CHIR99021 (CHIR). All cells were cultured in the presence of 1 μM QVD to exclude caspase-mediated Mcl-1 breakdown. After 24 h Mcl-1 expression was assessed by flow cytometry (n = 3). Values show means ± sem. GMI = Geometric Mean Intensity *P < 0.05, **P < 0.01, ***P < 0.001 (ANOVA with Bonferroni’s post-testing).

In activated B cells BAFF mediated survival was shown to depend on PI3K signaling^19^,^20^. In addition, it is known that PI3K signaling can control Mcl-1 protein levels. Mcl-1 is targeted for proteasomal degradation following phosphorylation by GSK3β. PI3K phosphorylates and activates Akt, which in turn phosphorylates and inhibits GSK3β, thus promoting in Mcl-1 stabilization^21^. We tested on several levels of this signaling cascade whether it is responsible for the affinity-controlled survival B cells following BAFF stimulation. First, inhibition of PI3K signaling using a Pan-PI3K inhibitor or CAL-101, a specific PI3Kδ inhibitor, resulted in a concentration-dependent reduction in survival, both for high- and low-affinity B cells. Viability correlated closely with Mcl-1 protein levels (Fig. 4a,b). Second, in presence of equal amounts of BAFF, low affinity B cells demonstrated reduced capacity to phosphorylate Akt, even though it did not reach statistical significance (Fig. 4c). However, considering the major impact of Akt-inhibition on B cell survival (Fig. 4a,b), even small changes in Akt phosphorylation appear to have a large biological impact. This is possibly due to amplification of the signal downstream in the signaling cascade.

Third, B cells stimulated *in vitro* with low-affinity antigen demonstrated moderately reduced levels of pGSK3β. Low-affinity B cells stimulated with high amounts of BAFF, in contrast, showed increased levels of phosphorylated GSK3β, which closely correlated with Mcl-1 protein (Fig. 4d). Fourth, blocking GSK3 activity in antigen-stimulated B cells, increased Mcl-1 levels in a concentration-dependent fashion (Fig. 4e). In summary, Mcl-1 protein levels are regulated through the PI3K signaling cascade in activated B cells in response to BAFF. Low-affinity B cells are less responsive to BAFF and therefore have reduced capacity to induce the PI3K signaling cascade.

### BAFF signaling is limiting for survival after low-affinity triggering *in vivo*

**Figure 5:**
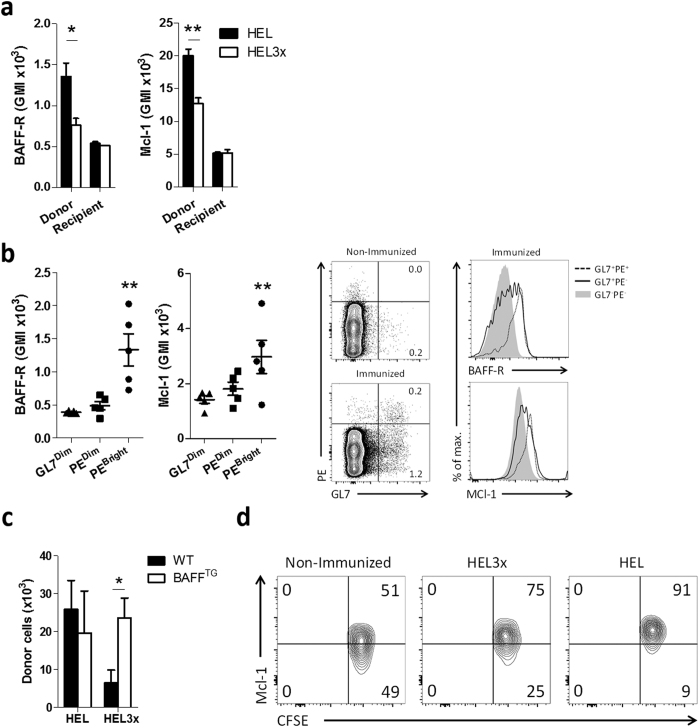
BCR affinity promotes BAFF-mediated Mcl-1 stabilization (**a**) 5 × 10^5^ purified HEL^TG^ B cells (Ly5.2) were injected in WT (Ly5.1) recipients. After 24 h, recipients were immunized with HEL/Ova or HEL3x/Ova. After 48 h, Mcl-1 and BAFF-R expression were assessed in donor and recipient B cells (n = 3). (**b**) Mice were immunized with R-Phycoerythrin (PE) in alum. After 6 days, splenic B cells were stained with PE. Mcl-1 and BAFF-R expression was analyzed in resting (GL7^Dim^) B cells, or in high (PE^Bright^) or low (PE^Dim^) affinity Germinal Center (GL7^Bright^CD38^Dim^) B cells. Representative flow cytometry plots of cells gated for B220^+^ is shown (n = 5). (**c,d**) 5 × 10^5^ purified CFSE-labeled HEL^TG^ B cells (Ly5.1/2) were injected in WT or BAFF^TG^ (both Ly5.2) recipients. After 24 h, recipients were immunized i.p. with HEL/Ova or HEL3x/Ova. After 48 h, cells were analyzed (**c**) The number of donor cells was quantified in spleen (n = 3–5). (**d**) Representative flow cytometry plots of B cells transferred to WT recipients and left non-immunized or immunized with HEL3x/Ova (HEL3x) or HEL/Ova (HEL) GMI = Geometric Mean Intensity. Gated is for B220^+^CD45.1^+^CD45.2^+^ cells. Values show means ± sem. *P < 0.05, **P < 0.01 (Student’s *t*-test and ANOVA with Bonferroni’s post-testing).

To confirm whether low-affinity B cells have reduced levels of the BAFF-R and Mcl-1 *in vivo*, HEL^TG^ B cells were transferred to congenic wild type (WT) recipients. Subsequently, mice were immunized with HEL-Ova or HEL3x-Ova and after two days, levels of Mcl-1 and BAFF-R in B cells were quantified by flow cytometry. Both HEL antigens increased Mcl-1 and BAFF-R expression compared to the bulk pool of recipient B cells, indicating activation of donor cells (Fig. 5a). In accordance with our *in vitro* observations, high-affinity B cells had increased levels of both Mcl-1 and BAFF-R compared to low-affinity cells (Fig. 5a). To demonstrate a similar regulation of BAFF-R and Mcl-1 in non-transgenic B cells, WT mice were immunized with R-Phycoerythrin (PE). The number of antigen-specific B cells is of very low frequency on day two after antigen injection. We therefore analyzed animals on day twelve after immunization. Since animals only received PE as an antigen, the majority of germinal center cells in spleen will be directed against this protein, which can be visualized by *ex vivo* staining with PE^6^. *Ex vivo* stained PE^Bright^ GC cells were considered to be high-affinity cells, whereas PE^Dim^ cells were of low-affinity. We found that both PE^Bright^ and PE^Dim^ germinal center (GL7^+^CD38^Dim^) B cells had higher levels of Mcl-1 and the BAFF-R compared to non-GC GL7^Dim^ cells. PE^Bright^ B cells had significantly higher levels of both molecules then PE^Dim^ germinal center B cells (Fig. 5b).

Our findings suggest that BAFF selectively promotes survival of high-affinity B cells. To test this directly, we made use of BAFF^TG^ mice which secrete high amounts of BAFF into the blood^22^. CFSE-labeled HEL^TG^ B cells were transferred in WT or BAFF^TG^ recipients which were subsequently immunized with HEL-Ova or HEL3x-Ova. Two days later donor cells were quantified in the spleen. WT and BAFF^TG^ mice contained equal numbers of high-affinity cells, but the number of low-affinity B cells was significantly increased in the presence of excess BAFF (Fig. 5c). Both immunogens had activated cells, as observed by induction of Mcl-1. Survival, rather than proliferation was responsible for differences in cell numbers, since cells did not yet show secondary peaks resulting from CFSE dilution (Fig. 5d). Of note, proliferation was readily induced on day 2 after stimulation *in vitro* (Fig. 1a). This difference is most likely the result from a delay in antigen encounter by B cells *in vivo*.

**Figure 6:**
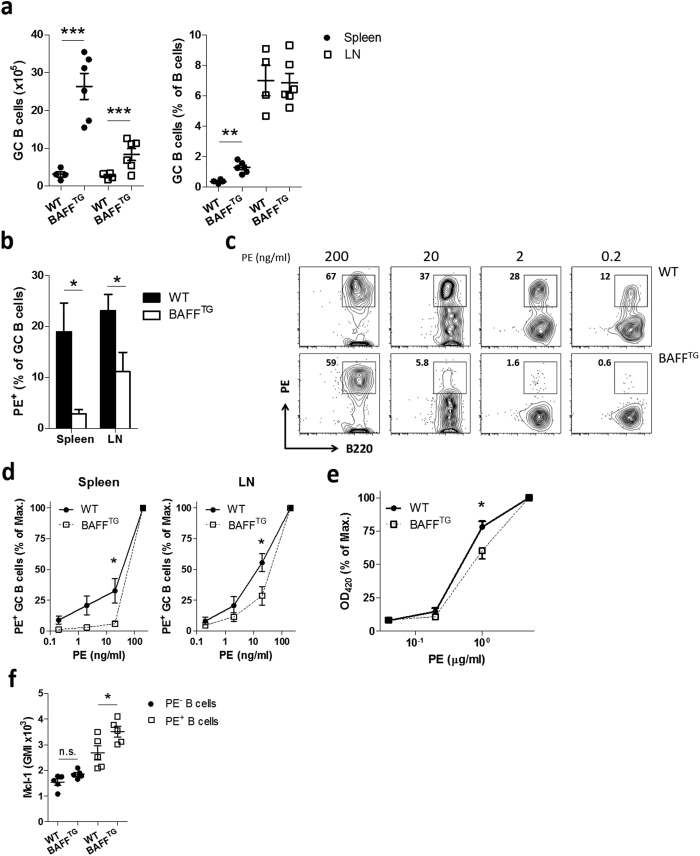
BAFF signaling limits survival after low-affinity triggering *in vivo*. (**a**) WT and BAFF^TG^ mice were immunized i.p. with TNP-KLH in alum. Relative and absolute Germinal Center (GC; B220^+^CD38^Dim^GL7^+^) cell numbers were quantified in spleen and draining Lymph node after 12 days (n = 4–6). (**b–f**) WT and BAFF^TG^ mice were immunized i.p. with PE in alum. After 12 days, mice were sacrificed and analyzed (n = 5). (**b**) The fraction of PE-binding B cells as a percentage of total GC B cells was determined by flow cytometry. (**c**) Germinal center B cells were stained *in vitro* with an increasing amount of PE and staining was visualized by flow cytometry. (**d**) quantification of data shown in **c**. PE-binding cells as a percentage of cells stained with 100 ng/ml PE (max.) is shown. (**e**) ELISA plates were coated with increasing amounts of PE. Antigen-binding IgG1 antibodies in serum were quantified. Values show means ± sem. (**f**) PE^+^ and PE^−^ B220^+^ B cells were analyzed for Mcl-1 expression by flow cytometry. *P < 0.05, **P < 0.01, ***P < 0.001 (Student’s *t*-test).

To assess the physiological impact of excess BAFF on B cell responses, WT and BAFF^TG^ mice were immunized with TNP-KLH and germinal center B cell numbers were quantified after two weeks. A significant increase of relative and absolute numbers of GC B cell numbers in BAFF^TG^ mice was observed in comparison to WT animals (Fig. 6a). BAFF^TG^ mice have two to three fold more follicular B cells in the spleen (data not shown), whereas the number of GC B cells was increased by 8–9 fold (8.4 fold ± 2.7). This indicates that the increased number of GC cells is not only the result of larger numbers of follicular B cells, but that BAFF availability is indeed limiting for activated B cell expansion *in vivo*. To investigate whether excess BAFF also impacts the affinity of the antigen-specific B cell pool, WT and BAFF^TG^ mice were immunized with PE. *Ex vivo* staining revealed that both in spleen and lymph nodes a smaller fraction of GC B cells was able to bind PE with high affinity (Fig. 6b). Antigen titration showed that total B cells from BAFF^TG^ mice more rapidly lost their ability to bind PE (Fig. 6c,d). This indicated that the antigen-specific B cell pool is of reduced overall affinity. In accordance with this observation, the PE-specific antibody response was also of reduced affinity (Fig. 6e), despite the fact that the total IgG pool is enlarged in these animals^22^. To investigate which molecule is responsible for this effect, PE^+^ B cells of WT and BAFF mice were sorted and expression of 40 pro- and anti-apoptotic moles was analyzed by MLPA. However, no significant differences were observed (Supplementary Figure 3). In contrast, Mcl-1 protein levels were significantly higher in PE^+^ cells of BAFF^TG^ mice (Fig. 6f).

Thus, BAFF availability was limiting for the survival of low-affinity B cells early after antigen stimulation. Immunization of mice with excess BAFF therefore reduced the average affinity of the antigen-specific B cell pool, most likely through specific induction of Mcl-1 protein.

### Noxa sets a survival threshold for high-affinity B cells *in vivo*

**Figure 7:**
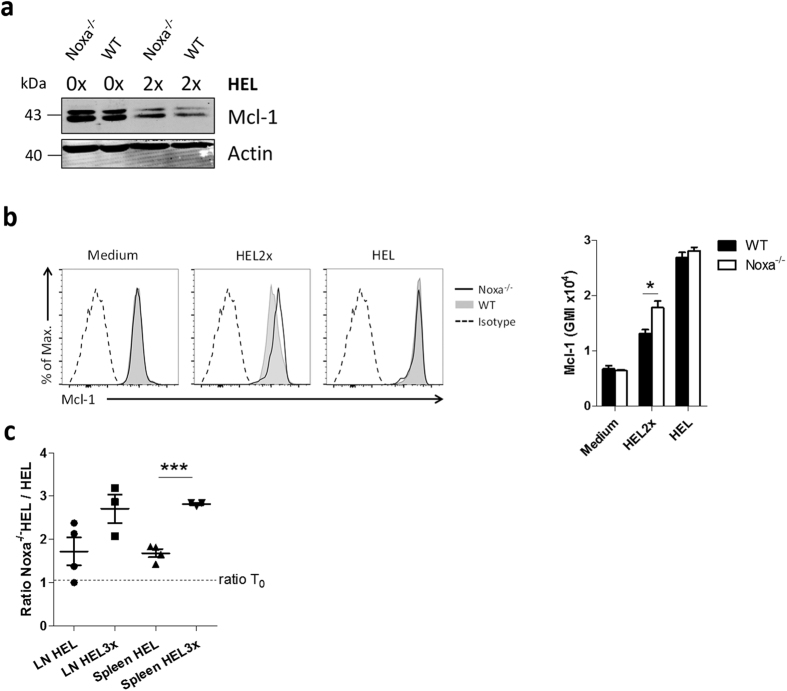
Noxa sets a survival threshold for high-affinity B cells. (**a,b**) HEL^TG^ (WT) or Noxa^−/−^HEL^TG^ (Noxa^−/−^) B cells were stimulated with 100 ng/ml BAFF in combination with 100 ng/ml HEL or HEL2x. After 48 h Mcl-1 protein levels were determined by (**a**) western blot (β-actin was used as a loading control) and (**b**) Flow Cytometry. (**c**) HEL^TG^ (Ly5.1/2) and Noxa^−/−^HEL^TG^ (Ly5.2) B cells were mixed in a 1:1 ratio and 5 × 10^5^ cells were injected in Ly5.1 WT recipients. After 24 h, recipients were immunized with HEL/Ova or HEL3x/Ova in alum. After 48 h donor cell ratios were determined in spleen and draining lymph node (n = 3–4). Dashed line indicates ratio between cells at time of injection. Values show means ± sem. *P < 0.05, ***P < 0.001 (Student’s *t*-test).

Finally, we investigated *in vivo* whether early survival of activated low-affinity B cells is mediated by Mcl-1. Since loss of even a single allele of Mcl-1 results in a dramatic loss of B cells^10^, we used mice deficient for its antagonist Noxa. Noxa^−/−^ mice have normal B cell development, but generate antibody responses of reduced affinity^6^. HEL^TG^ mice were crossed on a Noxa^−/−^ background and naive B cells from these animals were stimulated *in vitro* with HEL mutants. After high-affinity stimulation, Mcl-1 levels were comparable between WT and Noxa^−/−^ mice. However, after low-affinity stimulation, Noxa-deficient B cells contained higher levels of Mcl-1 compared to WT cells (Fig. 7a,b, Supplementary Figure S2d).

To demonstrate that Noxa-deficiency provides a specific survival advantage for low-affinity cells, HEL^TG^ B cells sufficient (Ly5.1/2) and deficient (Ly5.2) for Noxa were mixed in a 1:1 ratio and transferred to WT (Ly5.1) recipients. Subsequently, mice were immunized with HEL-Ova or HEL3x-Ova and the ratio between donor populations was determined two days later. In line with previous findings^6^, Noxa-deficient B cells had a slight survival advantage over WT cells in mice immunized with high-affinity antigens, as the ratio between cells increased by ~1.7 fold. Strikingly, upon low-affinity stimulation, the ratio between WT and Noxa^−/−^ donor B cells on average increased by almost three fold (Fig. 7c).

Thus, the Noxa/Mcl-1 axis controls survival of antigen-specific B cells and drives apoptosis of low-affinity B cells as a result of reduced access to BAFF compared to high-affinity B cells.

## Discussion

It has been shown previously that affinity-based B cell selection already takes place in the first days after antigen encounter, on the border between B and T cell regions^4^,^7^. However, how this earliest phase of selection was mediated mechanistically was unknown. Here we demonstrate that the affinity of BCR triggering controls the ability of B cells to respond to BAFF and that this is an important selection criterion for B cell clones in the first days after antigen encounter. BAFF promotes survival through stimulation of the PI3K signaling pathway and stabilization of the pro-survival protein Mcl-1. Low antigen-affinity results in insufficient access to BAFF and increases cell death upon immunization. Deficiency of Noxa, or excess availability of BAFF allows germinal center entry of more low-affinity clones, thus resulting in an overall reduction of the specificity of the antibody response^6^.

The role of BAFF in mature B cell survival has been studied extensively. Deficiency of BAFF or BAFF-R results in a more than tenfold reduction in peripheral B cells^23^,^24^ as T2 B cells become highly dependent on this cytokine for their survival. This effect is predominantly mediated through the NF-κB signaling cascade and T2 B cells deficient for IKK1 therefore demonstrate a phenotype that highly resembles that of BAFF^−/−^ mice^19^. Activated B cells further induce expression of the BAFF-R, which mediates survival through the PI3K signaling cascade^20^,^25^. In previous models, anti-IgM antibodies were used as an activating stimulus, which does not take the varying effect of antigen-affinity on BAFF-R expression levels into account. Rather, the ability of B cells to present antigen to follicular helper T cells in the germinal center and thus gain access to BAFF was therefore postulated as the way in which antigen-affinity is translated into a survival advantage^7^,^26^. However, BAFF expression is in fact relatively low in the germinal center^26^ and somatic hypermutation was only mildly affected in immunization models in which B cells do not have access to BAFF^26^. We show that the capacity of activated B cells to acquire sufficient amounts of BAFF through affinity-dependent BAFF-R induction is of immediate importance for their competitive fitness and controls the subsequent ability to seed germinal centers. In agreement with our findings, BAFF-induced PI3K signaling was shown to be crucial for IgM production upon immunization, whereas IgG1 levels were less dependent on this signaling cascade^19^. In absence of BAFF, deficiency of the PI3K phosphatase PTEN results in an increase of germinal center size, which is predominantly the result of increased numbers of IgM-producing cells and reminiscent of an increase in the number of GC-seeding cells^19^.

Studies using mice deficient for various pro- and anti-apoptotic molecules also indicate a differential control of activated B cell viability before and after germinal center formation. Noxa deficiency does not affect somatic hypermutation upon immunization, but is associated with an increase of germinal center size and reduced antibody affinity as a result of increased numbers of GC-seeding clones^6^. Mice lacking one allele of Mcl-1 have a strong reduction in the number of germinal center B cells, but affinity-based selection in the remaining cells is not affected^10^. In contrast, deficiency of Bim or Puma, two pro-apoptotic proteins which are induced in germinal center B cells, results in a strong reduction of clones carrying somatic hypermutations and an increase in antigen forming cells, whereas relative germinal center size is not affected^12^,^27^. Bcl-XL appears to play its role much later in the B cell response, when antigen-experienced plasmablasts enter circulation^10^.

The affinity-dependent capacity of B cells to access BAFF in order to stabilize Mcl-1, guarantees that in each B cell response only the cells of highest affinity survive, independent of the absolute level of antigen-affinity. Interestingly, CD8 T cells use a similar mechanism to select for high affinity clones. CD8 T cell receptor affinity correlates with expression of the IL-2 receptor through affinity-dependent induction of the transcription factor T-bet^28^. IL-2, in turn, promotes proliferation and stabilization of Mcl-1^15^. High-affinity T cells therefore have a selective advantage over low-affinity cells in their competition for limiting amounts of IL-2. Mice deficient for Noxa have a reduction in the overall affinity of the effector CD8 T cell pool, though the magnitude of both responses is unaffected^6^,^15^. We observed that BCR-affinity also positively correlated with expression of CD25 in B cells. In our system IL-2 did not play a role in B cell survival, possibly as a result of differences in the use of PI3K and Stat5 isoforms between B and T cells^29^,^30^. Nevertheless, for both B and T cells, affinity-sensing of the antigen-responsive pool is mediated by regulation of the Noxa/Mcl-1 axis through differential cytokine receptor expression, thus establishing a common theme.

Surprisingly, mice deficient for BAFF have been reported to form equal numbers of germinal centers as wild type mice in the first week after immunization^23^,^24^. This apparently contradicts our model of BAFF as an essential molecule for survival of pre-GC B cells. However, since BAFF^−/−^ mice have a strong reduction in mature B cell numbers, the cells that do survive in these animals therefore have abundant access to all other pro-survival factors but BAFF. Moreover, it seems likely that these cells have altered expression levels of pro-survival Bcl-2 family members in order to sustain their vitality in absence of BAFF. Of course, similar limitations apply to our *in vivo* models that make use of BAFF^TG^ animals. Mice with excess BAFF have reduced negative B cell selection and therefore show B cell hyperplasia from the T2 stage onwards^22^. Increased numbers of antigen-responsive B cells upon immunization may therefore be partially the result of an increase in the broadness of the B cell repertoire. However, HEL^TG^ B cells also showed increased survival after immunization with low-, but not high-affinity antigens when transferred to BAFF^TG^ animals. Thus, BAFF appears to be important for shaping both the naïve and early antigen-responsive B cell repertoire.

In summary, we here uncover a previously unknown link between BCR affinity and BAFF receptor signaling in early B cell survival and selection. Our findings may have important implications for immunization strategies that aim to restrict or broaden the scope of the protective antibody response.

## Methods

### Mice

Animals were used at 6–12 weeks of age, age- and sex-matched within experiments and were handled in accordance with institutional and international guidelines. Wild type C57BL/6 (B6, strain 00664), HEL^TG^ (strain 002595) and B6 Ly5.1 (strain 002014) mice were purchased from the Jackson Laboratories and kept as breeding colonies in our local animal facility. Noxa^−/−^ mice were a gift from Dr. A. Strasser (WEHI, Melbourne). BAFF^TG^ mice were described previously^22^, and were a gift of Biogen Idec. All mice were either generated in B6 mice or backcrossed at least ten times on this background. All animal experiments were performed after approval of our institute’s animal ethics committee (Dierexperimentencommissie AMC). All methods were performed in accordance with national and international guidelines and regulations.

### Cell culture

Splenic HEL^TG^ B cells were purified to >95% purity using the MACS cell separation system (Myltenyi) and anti-CD19 microbeads (Myltenyi). Cells were cultured in RPMI with 10% fetal calf serum (FCS) with HEL proteins that were purified as described^13^. Cells were cultured in combination with indicated cytokines (R&D Systems), LPS (Sigma-Aldrich) or anti-CD40 (Bioceros). For pAkt stainings, cells were deprived from stimuli for 3 h in medium with 1% FCS, followed by stimulation with 500 ng/ml BAFF. Ly294002 (Invitrogen) and CAL-101 (apexbio) were used to inhibit PI3K signaling. Cell division was analyzed by CFSE (Molecular Probes) dilution. To generate 3TBAFF cells, the intracellular and transmembrane domains of CD40L (amino acids 0–112 of NP_000065.1) were fused by overlap extension PCR including a spacer (N-PAAAAAASAAAAAAWVPVAT-C) to the extracellular domain of BAFF (amino acids 133–285 of NP_006564.1) and introduced in the LZRS vector. Generation of retroviral particles and transductions were done as described^31^. Cells were selected based on highest GFP expression using two rounds of cell sorting. Functional BAFF expression was assessed as described using JTF cells^32^. Cells were gamma-irradiated (30Gy) and plated out 24 h before co-cultivation with B cells.

### Immunization and transfer

Mice were immunized intraperitoneally with 50 μg Phycoerythrin (PE; Sigma-aldrich), HEL/OVA (1:1), HEL3x/OVA (1:1) or TNP-KLH (Biosearch technologies) in Alum (Imject Alum; Thermo Fisher Scientific) as described^6^,^13^. For adoptive transfer, MACS-purified cells (2*10^6^ for analysis on day 2, 5*10^4^ for analysis on day 6–12) were injected in congenic recipients.

### Flow cytometry

Single-cell suspensions were obtained by mincing the specified organs through 40 μm cell strainers (Becton Dickinson). Erythrocytes were lysed with an ammonium chloride solution (155 mM NH4Cl, 10 mM KHCO3, and 1 mM EDTA) and cells were subsequently counted using an automated cell counter (SCHÄRFE SYSTEM). Cells (~10^6^) were collected in PBS with 0.5% bovine serum albumin (Sigma-Aldrich) and stained for 30 min at 4 °C with antibodies in the presence of anti-CD16/CD32 (clone 2.4G2, a gift of Louis Boon, Bioceros). Where possible, a viability dye was added to exclude dead cells (PI (Molecular Probes), ToPro3 (Molecular Probes), Fixable Viability Dye (eBioscience)). Monoclonal antibodies against B220 (6B2), CD38 (90), GL7 (GL7), CD19 (eBio1D3), IgM (eB121-15F9), CD268 (eBio7H22-E16) and CD25 (PC61.5) were purchased from eBioscience, CD124 (mIL4R-M1) and TACI (1A1) from BD Bioscience and BCMA (161616) from R&D Systems. Fixation and permeabilization of cells for intracellular Mcl-1 (Rockland Immunochemicals) staining was performed using the BD Fix/Perm kit. For pAkt staining, cells were fixed with 2% paraformaldehyde, permeabilized in 90% methanol, and stained with fluorescently labeled pAkt^T308^ (Cell Signaling). Antigen-specific B cells were visualized by *ex vivo* labelling using PE (Sigma-Aldrich). FACS experiments were performed on a FACSCalibur or FACSCanto (Becton Dickinson) and analyzed with FlowJo software (TriStar). Doublets were excluded from analysis by gating based on FSC-A vs. FSC-H and SSC-A vs. SSC-H. Positive gating was based on stainings with appropriate isotype controls (eBioscience).

### RT-MLPA and RT-PCR analysis

Total RNA was extracted using the trizol isolation method (Invitrogen). mRNA levels of apoptosis genes were analyzed with the Mouse RT-MLPA kit (MRC-Holland)^33^ according to the manufacturer’s instructions. cDNA was generated using oligodeoxythymidine (oligo dT) and Superscript II Reverse Transcriptase (Invitrogen). Analysis of RNA transcripts were amplified by polymerase chain reaction (PCR) using the primer-pair TGGACATACAAGCAGCCTGG/TTTTCCAGGGACTC TTGCTGG (CD268). 18S was used as internal control (TCAAGAACGAAAGTCGGAGG/GGACATCTAAGG GCATCACA).

### Immunoblot and ELISA

For immunoblot cells were lysed in Laemmli lysis buffer at the indicated time points after stimulation containing protease and phosphatase inhibitors (Roche). SDS-PAGE gel electrophoresis was performed using the Bio-Rad mini-PROTEAN electrophoresis system with primary antibodies against β-Actin (Santa Cruz Biotechnology), Bim (Stressgen), Bcl-XL (Transduction Laboratories), Bcl-2 (Alexis Biochemicals), Mcl-1 (Rockland), Bax (BD Pharmingen) and pGSK3β (Santa Cruz). Binding was visualized using IRDye 680 or 800 labeled secondary antibodies and an Odyssey Imager (Li-Cor). Quantification of signal was performed using Odyssey 3.0 software. For ELISA, Maxisorb plates (NUNC) were coated O/N with PE (Sigma-Aldrich). Antibody levels were determined using biotinylated detection antibodies (Southern Biotech), streptavidin-labeled alkaline phosphatase (Southern Biotech) and SigmaFAST pNPP tablets (Sigma-Aldrich). Optical density was determined with a photo-spectrometer (BioRad).

### Statistical analysis

Statistical analysis of the data was performed using the unpaired Student’s t-test, Wilcoxon rank-sum test or ANOVA with Bonferroni’s post testing where applicable. Asterisks denote significant differences (*p < 0.05, **p < 0.01, ***p < 0.001).

## Additional Information

**How to cite this article**: Wensveen, F. M. *et al.* Antigen-affinity controls pre-germinal center B cell selection by promoting Mcl-1 induction through BAFF receptor signaling. *Sci. Rep.* **6**, 35673; doi: 10.1038/srep35673 (2016).

## Supplementary Material

Supplementary Information
