# Erratum: Antigen-affinity controls pre-germinal center B cell selection by promoting Mcl-1 induction through BAFF receptor signaling

**DOI:** 10.1038/srep39366

**Published:** 2017-01-12

**Authors:** Felix M. Wensveen, Erik Slinger, Martijn HA van Attekum, Robert Brink, Eric Eldering

Scientific Reports 6: Article number: 3567310.1038/srep35673; published online: 10 20 2016; updated: 01 12 2017

The original version of this Article contained an error in the title of the paper, where the word “center” was incorrectly given as “censter”. This has now been corrected in the PDF and HTML versions of the Article.

