# Supplementary material for: Antigen-affinity controls pre-germinal centser B cell selection by promoting Mcl-1 induction through BAFF receptor signaling
Source: Sci Rep. 2016 Oct 20;6:35673. doi: 10.1038/srep35673 (PMC5071843; doi:10.1038/srep35673)
Supplement: Supplementary Information [file srep35673-s1.doc]

**Antigen-affinity controls pre-germinal center B cell selection by promoting Mcl-1 induction through BAFF receptor signaling**

Felix M. Wensveen^1*^, Erik Slinger^1^, Martijn HA van Attekum^1^, Robert Brink^2^, Eric Eldering^1^

^1^Department of Experimental Immunology, Academic Medical Center, 1105AZ, Amsterdam, The Netherlands

^2^Immunology Division, Garvan Institute of Medical Research, NSW 2010, Darlinghurst, Australia

Correspondence:

Felix Wensveen

Dept Experimental Immunology, room K0-144

Meibergdreef 9

1105AZ Amsterdam the Netherlands

*Email; Felix.Wensveen@gmail.com

**Figure S1. BAFF potently promotes survival of antigen-stimulated B cells.** (**a**) HEL^TG^ B cells were stimulated with 100ng/ml HEL in combination with the following B cell stimuli; BAFF (3, 16, 80, 400 ng/ml), APRIL (3, 16, 80, 400 ng/ml), IL-6 (3, 16, 80, 400 ng/ml), αCD40 (0.08, 0.4, 2, 10 µg/ml) or LPS (0.008, 0.04, 0.2, 1 µg/ml). (n=3) and survival was determined after 48h by PI staining using flow cytometry (**b**) The data from Figure 1a were replotted as percentage of the maximal signal to allow direct comparison of activation, proliferation and viability (n=3). (**c**) Schematic of the BAFF-construct used to generate 3TBAFF cells (drawn to scale). (**d**) qPCR analysis of 3T3 cells stably transfected with BAFF (3TBAFF) or empty vector (3TEV).Shown are mean BAFF expression levels ±SD compared to loading control HPRT of an *in triplo* qPCR experiment. (**e**) BAFF-reporter Jurkat-TACI:FAS cells that undergo apoptosis upon BAFF signaling^32^ were cultured on 3TEV or 3TBAFF cells . After 24h, the percentage of living cells (Dioc6-) was determined by flow cytometry. Values show means ± sem. * P<0.05 (Student’s *t*-test). GMI=Geometric Mean Intensity.

**Figure S2. BAFF controls Mcl-1 protein levels in a BCR affinity-dependent manner. a-c** Quantification of blots shown in figure 3. Protein levels were quantified for all samples using densitometry, normalized for β-actin and shown as fold induction relative to values at 0h. (**a**) HEL^TG^ B cells were stimulated with 100ng/ml HEL, HEL2x or HEL3x in the presence 100ng/ml BAFF. (n=8) (**b**) HEL^TG^ B cells were stimulated for 24h with 100ng/ml HEL, 100ng/ml BAFF or both (n=3) (**c**) HEL^TG^ B cells were stimulated with 100ng/ml HEL, HEL2x or HEL3x in the presence or absence of 100ng/ml BAFF (n=3). (**d**) HEL^TG^ B cells were stimulated with 100ng/ml HEL, HEL2x or HEL3x in the presence 100ng/ml BAFF or with 100ng/ml HEL3x in combination with 500ng/ml BAFF (HiBaff). Total cell lysates were then probed by western blot for the indicated proteins. β-Actin was used as a loading control. Quantification of Bim shows cumulative signal for the XL, L and S isoforms. (**d**) HEL^TG^ (WT) or Noxa^-/-^HEL^TG^ (Noxa^-/-^) B cells were stimulated with 100ng/ml BAFF in combination with 100ng/ml HEL or HEL2x. After 48h Mcl-1 protein levels were determined by western blot. Quantification of blots from figure 7a shows Mcl-1 signal normalized for β-actin.

**Figure S3. PE^+^ B cells in BAFF^TG^ mice do not show differences in expression levels of apoptotic proteins.** WT and BAFF^TG^ mice were immunized i.p. with PE in alum. After 12 days, mice were sacrificed and PE^+^ cells were sorted from isolated splenocytes. cDNA isolated from these cells was analyzed by MLPA for expression of 40 pro- and anti-apoptotic proteins. Peaks represent signals relative to the sum of all peaks combined (n=5).
